# Parkinsonian phenotype in Late-onset pantothenate kinase-associated neurodegeneration: a case report

**DOI:** 10.1007/s13760-025-02906-9

**Published:** 2025-10-10

**Authors:** Faustine Lebout, Vandernoot Isabelle, Desmyter Laurence, Gil Leurquin-Sterk, Virginie Destrebecq

**Affiliations:** 1 Department of Neurology, Hôpital Erasme, Hôpital Universitaire de Bruxelles, Université Libre de Bruxelles, Brussels, Belgium; 2 Department of Genetics, Hôpital Erasme, Hôpital Universitaire de Bruxelles, Université Libre de Bruxelles, Brussels, Belgium; 3 Departement of Nuclear Medicine, Hôpital Erasme, Hôpital Universitaire de Bruxelles, Université Libre de Bruxelles, Brussels, Belgium

## Introduction

Pantothenate kinase-associated neurodegeneration (PKAN) is an autosomal-recessive disorder caused by defects in the pantothenate kinase 2 protein, encoded by the *PANK2* gene. It’s the most common form of neurodegeneration with brain iron accumulation (NBIA) [1]. 

Traditionally, the disease is divided into two types of presentation: The classic PKAN, characterized by childhood onset of dystonia, gait abnormalities, and clumsiness due to lower extremity dystonia, along with mild global developmental delay, pigmentary retinopathy and dyspraxia. The atypical PKAN refers to a later-onset form, usually encountered in the second and third decade of life, and features a more insidious progression with predominant parkinsonian symptoms, chorea, tremor, neuropsychiatric and speech disorders [1]. 

We here report a very late-onset, genetically confirmed, case of PKAN starting at 58 years of age presented as a Parkinsonian gait phenotype without cognitive decline, dystonia or neuropsychiatric features contributing to the expansion of its known clinical spectrum.

## Case report

A 69-year-old woman presented with a progressive gait impairment beginning at 58 years of age with a walk with small steps, festination, falls and writing and speech difficulties. There were no neuropsychiatric features, no cognitive decline (Montreal Cognitive Assessment version 8.1 of 29/30), no dystonia and no relevant family history. After 2–3 years of initial worsening, the symptomatology was reported as stable by the patient. The complaints were independent of any infectious context or pyrexia. At initial presentation, the patient had been treated with levodopa-benserazide (1100 mg/day of levodopa) and pramipexole (1.05 mg/day) during the past 12 months by another center but with any significant benefit, leading to discontinuation of both medications.

The neurological examination revealed mild hypophonia, dysarthria, and a Parkinsonian gait characterised by a forward-leaning posture, small steps, mild festination, gait rigidity, and extrapyramidal rigidity in all four limbs. There was no chorea, dystonia, tremor or marked bradykinesia, but we noted an impairment of alternating rapid hand movements with interruptions, hesitations and perseveration (video attached). 

**Fig. 1:**
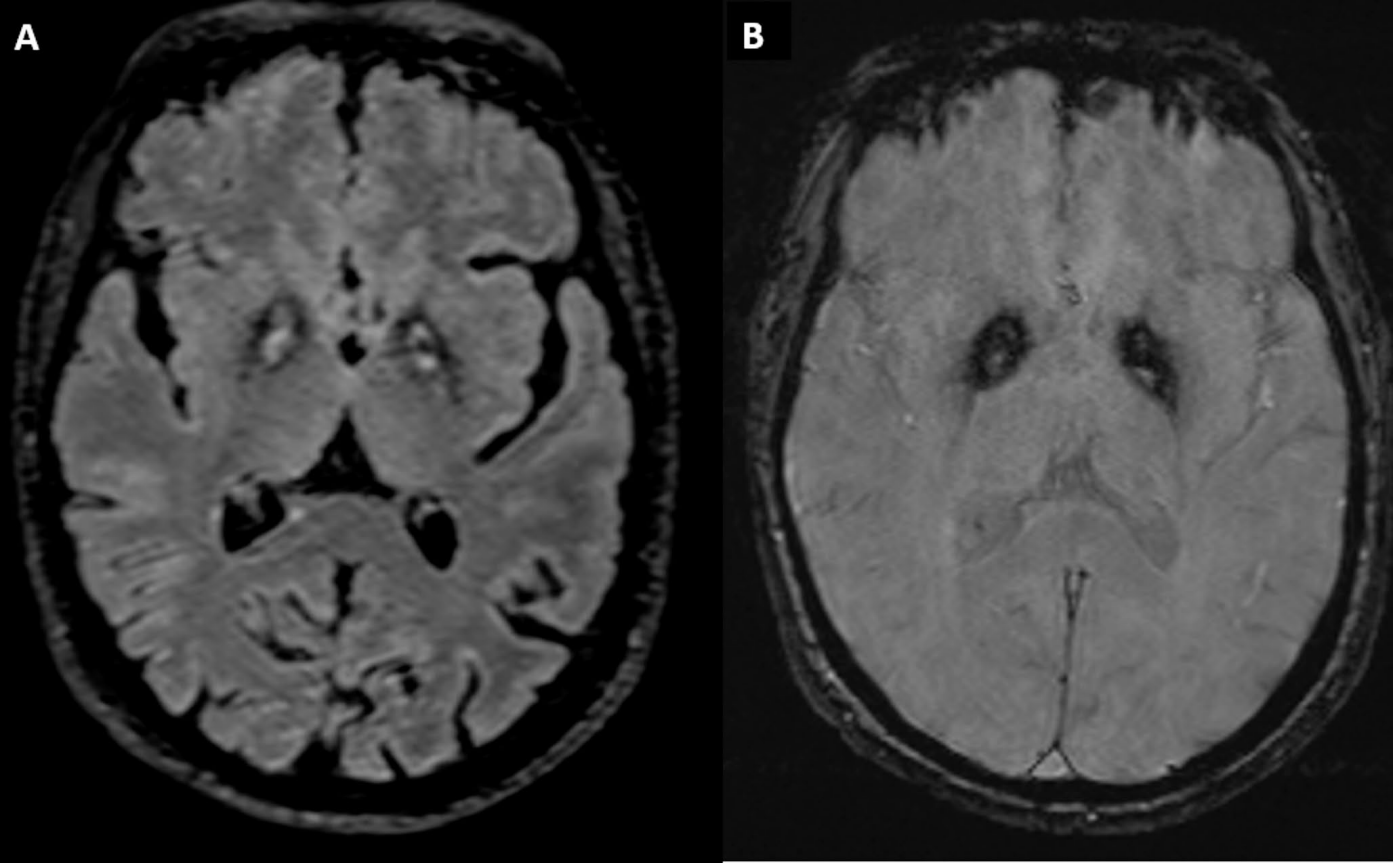
Brain MRI Imaging. (**A**) Axial-slice of T2-FLAIR weighted sequences with the classical “Eye of the tiger sign” defined by a bilateral central region with hyperintense signal surrounded by hypointensity in the globus pallidus. (**B**) Axial slice of Susceptibility Weighted Imaging sequences showing susceptibility artifact (low signal) in corresponding areas from iron deposition

**Fig. 2:**
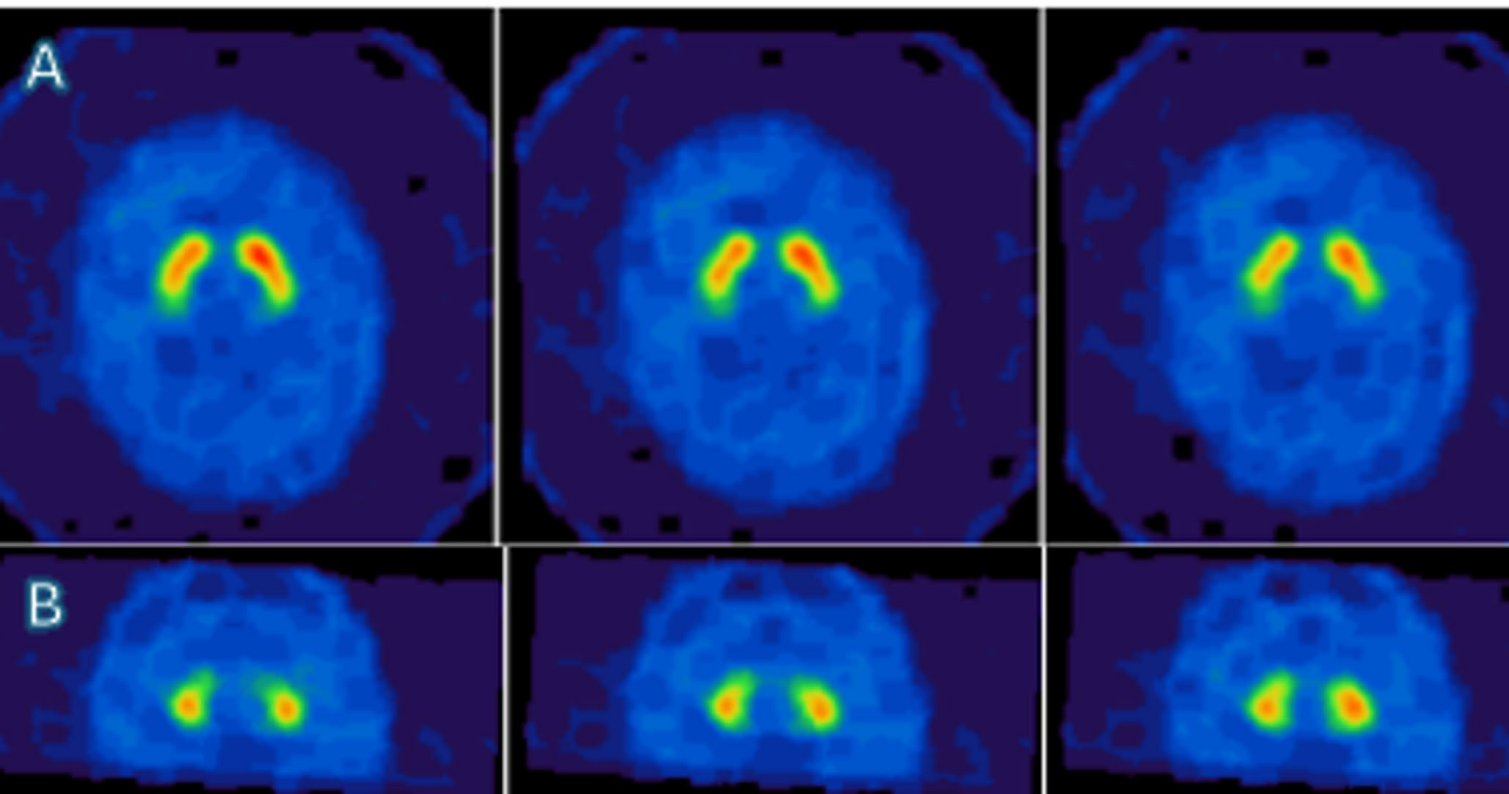
The quantitative indices (specific binding ratios) of I-123 ioflupane single-photon emission computed tomography (DATscan) were considered as normal. Discriminate value reported in litterature to differenciate essential tremor/healthy subjects and parkinsonism disease are above 0.76 for putamen (Se 87%, Spe 85%) and above 1,56 for caudate nuclei (Se 82%, Spe 85%).^2^ (**A**) Axial slice shows normal uptake of dopamine in both putamen with respectively specific binding ratios at 2.1 (right putamen) and 1.65 (left putamen). (**B**) Coronal slice shows normal captation in both caudate nuclei with specific binding ratios at 2.08 (right caudate nucleus) and 2 (left caudate nucleus)

Brain magnetic resonance imaging (MRI) showed an “eye of the tiger” sign, defined by a bilateral central region with hyperintense signal surrounded by hypointensity in the globus pallidus on T2-weighted sequences (Fig. 1) [1]. There was no evidence of cerebellar or cortical atrophy. Fluorodeoxyglucose positron emission tomography (FDG-PET) and I-123 ioflupane single-photon emission computed tomography (DATscan) were normal, in accordance with previous cases reported (Fig. 2) [2, 3].

Based on the observation of iron deposition and late-onset symptomatology, NBIA disorder was suspected and a whole exome sequencing using a Next-Generation Sequencing gene panel was performed including 73 genes involved in NBIA diseases or related conditions. During the following year, neurological symptoms remained stable, however the patient developed bilateral gonalgia, further impairing her gait and necessitating bilateral total knee arthroplasty. No neuropsychiatric features and no cognitive impairment were observed.

Finally, genetic testing confirmed a homozygote missense variant (NM_153638.4: c.1280G > C p.Gly427Ala) in the *PANK2* gene. This variant has not been previously reported in the literature and is absent from the gnomADv4 control database (gnomad.broadinstitute.org) and most prediction tools predict it to have a deleterious effect. Considering this, and the fact that the variant is located in a region where other mutations have already been reported, as well as the good correlation with the phenotype, this variant was classified as likely pathogenic. Family segregation analysis could not be performed as both patient’s parents were deceased at the time of assessment.

## Discussion

We report here a very-late onset, genetically confirmed, case of PKAN with predominent Parkinsonian gait feature in the absence of dystonia, chorea, cognitive impairment or neuropsychiatric features. This case could mimic a “pure” motor axial parkinsonism phenotype and expands the recognized clinical spectrum of this disease. As illustrated by this case, the response to levodopa is poor or absent [4]. 

Neurological deterioration in PKAN has traditionally been described as occurring between the second and third decades, and, to date, only 17 cases were reported in the literature with symptom onset after 50 years of age [3]. Based on previous genetically confirmed cases, late-onset PKAN is associated with more insidious and pauci-symptomatic course, mainly characterized by parkinsonian symptoms, chorea and gait disturbances [3]. This contrasts with earlier atypical PKAN which is usually also associated with prominent neuropsychiatric features, spasticity and other movement disorders such as dystonia [1]. 

The MRI characteristic pattern with the “eye of the tiger” sign in the globus pallidus is pathognomonic of PKAN disease [1]. 

Missense variants are the most common type of mutation identified in both typical and atypical PKAN forms but, to our knowledge, this specific variant (NM_153638.4: c.1280G > C p.Gly427Ala) has never been described in literature. Interestingly, this mutation is located within the same functional region as other NBIA-related mutations such as p.Leu425Pro, Leu425His, p.Leu424Phe which are associated with “classical” pediatric presentations[5].

Consistently with previous late-onset PKAN case reported, this case challenges the traditionally dichotomous classification of this condition and contributes to expanding the phenotypic spectrum of PKAN. It highlights the importance of considering PKAN in older patients with progressive Parkinsonian gait phenotype.

## Supplementary Information

Below is the link to the electronic supplementary material.

Supplementary Material 1

Supplementary Material 2

## Data Availability

No datasets were generated or analysed during the current study.

## References

[1] Pohane MR, Dafre R, Sontakke NG (2023) Diagnosis and treatment of pantothenate kinase-associated neurodegeneration (PKAN): a systematic review. Cureus 15:e46135 37900501 10.7759/cureus.46135 PMC10612532

[2] Nichols KJ, Chen B, Tomas MB, Palestro CJ (2018) Interpreting 123I-ioflupane dopamine transporter scans using hybrid scores. Eur J Hybrid Imaging 2(1):10 29855627 10.1186/s41824-018-0028-0 PMC5960650

[3] Sipilä JOT, Hietaharju A, Saukkonen AM (2025) Very late-onset neurodegeneration with brain iron accumulation associated with mild chorea: a clinicopathological case. Mov Disord Clin Pract 12:835–841 40079804 10.1002/mdc3.70032 PMC12187956

[4] Feuerstein J, Olvera C, Fullard M (2020) Treatment responsiveness of parkinsonism in atypical pantothenate kinase-associated neurodegeneration. Mov Disord Clin Pract 7(Suppl 3):S71–S73 33015228 10.1002/mdc3.13056 PMC7525193

[5] Zhou J, He J, Kou LP (2017) Phenotypic and genotypic features of twenty children with classic pantothenate kinase-associated neurodegeneration. Zhonghua Er Ke Za Zhi 55:678–682 28881514 10.3760/cma.j.issn.0578-1310.2017.09.011

