# Supplementary material for: Parkinsonian phenotype in Late-onset pantothenate kinase-associated neurodegeneration: a case report
Source: Acta Neurol Belg. 2025 Oct 10;125(6):1715–8. doi: 10.1007/s13760-025-02906-9 (PMC12644214; doi:10.1007/s13760-025-02906-9)
Supplement: Supplementary file 1 — Supplementary Material 1 [file 13760_2025_2906_MOESM1_ESM.pdf]

Consent for Publication of Photos, Videos, and Other Identifying Materials

I authorize publication of photographs, video tapes, sound recordings, or other materials that may identify my child or me. I understand that I will not own the copyright to these materials and grant permission for use of these materials for teaching, research, scientific meetings, other professional journals, medical books, broadcasts, advertising, and other purposes. These materials may appear in print and online and the public may have access to them.

The opportunity to view the materials and the manuscript containing them has been offered to me. I have either reviewed them or chosen not to review them.

HERNÁIZ MARÍA Hernáiz
____

Signature and printed name of patient

OR

______________________________ ______________________________

Signature & printed name of guardian Relationship to patient

5 juny 2025
____

Date
